# Giant Masses in a Non-Cirrhotic Liver: All That Look Epithelial Are Not Carcinoma

**DOI:** 10.7759/cureus.110020

**Published:** 2026-06-01

**Authors:** Dipanwita Biswas, Suvradeep Mitra, Aravind Sekar, Swapnesh Sahu, Lileswar Kaman

**Affiliations:** 1 Histopathology, Postgraduate Institute of Medical Education and Research, Chandigarh, IND; 2 General Surgery, Postgraduate Institute of Medical Education and Research, Chandigarh, IND

**Keywords:** hepatic angiomyolipoma, histopathology, immunohistochemistry, liver, pecoma

## Abstract

Angiomyolipoma (AML) is an uncommon mesenchymal neoplasm with myomelanocytic differentiation. Hepatic AML (HAML) is an uncommon tumor that often mimics a primary hepatic epithelial malignancy and is a challenging diagnosis for clinicians and pathologists. This article discusses the clinical, radiological, histopathological, and immunohistochemical details of two cases of HAML and their differential diagnoses. A 46-year-old woman and a 38-year-old man presented with pain, weakness, and weight loss. Radiology revealed large, well-defined, heterogeneous, enhancing masses in the non-cirrhotic liver. Histopathology showed a variable combination of epithelioid cells, spindle cells, adipocytes, and thick-walled blood vessels with smooth muscle actin (SMA) and HMB45 immunopositivity. The diagnosis of HAML depends on classical histopathology. However, a wide array of differential diagnoses should be considered depending on the proportionate representation of each component. A detailed histological evaluation, judicious use of immunohistochemistry to document myo-melanocytic differentiation, and awareness of the entity are useful for the correct diagnosis.

## Introduction

Angiomyolipoma (AML) is a mesenchymal neoplasm with myomelanocytic differentiation and is a subtype of PEComas (perivascular epithelioid cell tumors) [1,2]. The PEComa family comprises lymphangioleiomyomatosis, clear-cell "sugar" tumor, clear-cell myomelanocytic tumor of the falciform ligament/ligamentum teres, and diverse other tumors in addition to AML [3]. It is composed of an admixture of smooth muscle (myo), mature adipocytes (lipo), and vessels (angio) in variable proportions. The kidney is the most common site of AML and can be associated with tuberous sclerosis [4]. In contrast, hepatic angiomyolipoma (HAML) is uncommon and mostly sporadic [1,2,4]. Since its initial description in 1976, the diagnosis of HAML has remained a challenge for clinicians and radiologists owing to its nonspecific features, and the diagnosis depends on histopathology [4]. However, the histopathological diagnosis of HAML is not straightforward because of its rarity and the admixture of components in variable proportions, leading to diverse morphological patterns. In addition, HAML often exhibits an epithelioid morphology simulating more common hepatic tumors, such as hepatocellular carcinoma, sarcomatoid hepatocellular carcinoma, epithelioid hemangioendothelioma, metastatic gastrointestinal stromal tumor, leiomyosarcoma, and other tumors with epithelioid/sarcomatoid morphology [5,6]. We report two cases of HAML in a 46-year-old woman and a 38-year-old man, along with their clinicopathological features and differential diagnoses.

## Case presentation

Case 1

A 46-year-old woman presented with right upper quadrant pain for nine months associated with nausea, easy fatigability, and weight loss. She had anemia, and her serum α-fetoprotein (AFP), carcinoembryonic antigen (CEA), and CA-19-9 levels were normal (Table 1). She had markedly elevated levels of protein induced by vitamin K absence or antagonist-II (PIVKA-II).

**Table 1:** Various hematological and biochemical parameters along with the normative values AST: Aspartate aminotransferase; ALT: Alanine aminotransferase; ALP: Alkaline phosphatase; INR: International normalized ratio; PIVKA-II: Protein induced by vitamin K absence or antagonist-II; CEA: Carcinoembryonic antigen.

Parameters	Normal range	Case 1	Case 2
Hemoglobin (gm/dL)	12-14	8	11.3
Total leukocyte count (/µL)	4000-11000	6570	4880
Platelet (/µL)	1,50,000-4,50,000	2,10,000	2,47,000
INR	0.8-1.1	0.9	1.1
Urea (mg/dL)	10-50	15	32
Creatinine (mg/dL)	0.5-1.2	0.7	0.52
Total bilirubin (mg/dL)	0.1-1.2	1.1	0.7
Direct bilirubin (mg/dL)	<0.3	0.3	0.3
AST (U/L)	2-40	12	22
ALT (U/L)	2-41	20	45
ALP (IU/L)	42-128	84	144
Total protein (g/dL)	6.4-8.3	7.7	7.7
Albumin (g/dL)	3.4-4.8	4	4.3
α-fetoprotein (ng/mL)	0-40	5.5	2
PIVKA-II (mAU/L)	10-40	17235	14
CEA (ng/mL)	0-2.5	0.2	0.2
CA-19-9 (U/mL)	0-35	0.6	1

**Figure 1:**
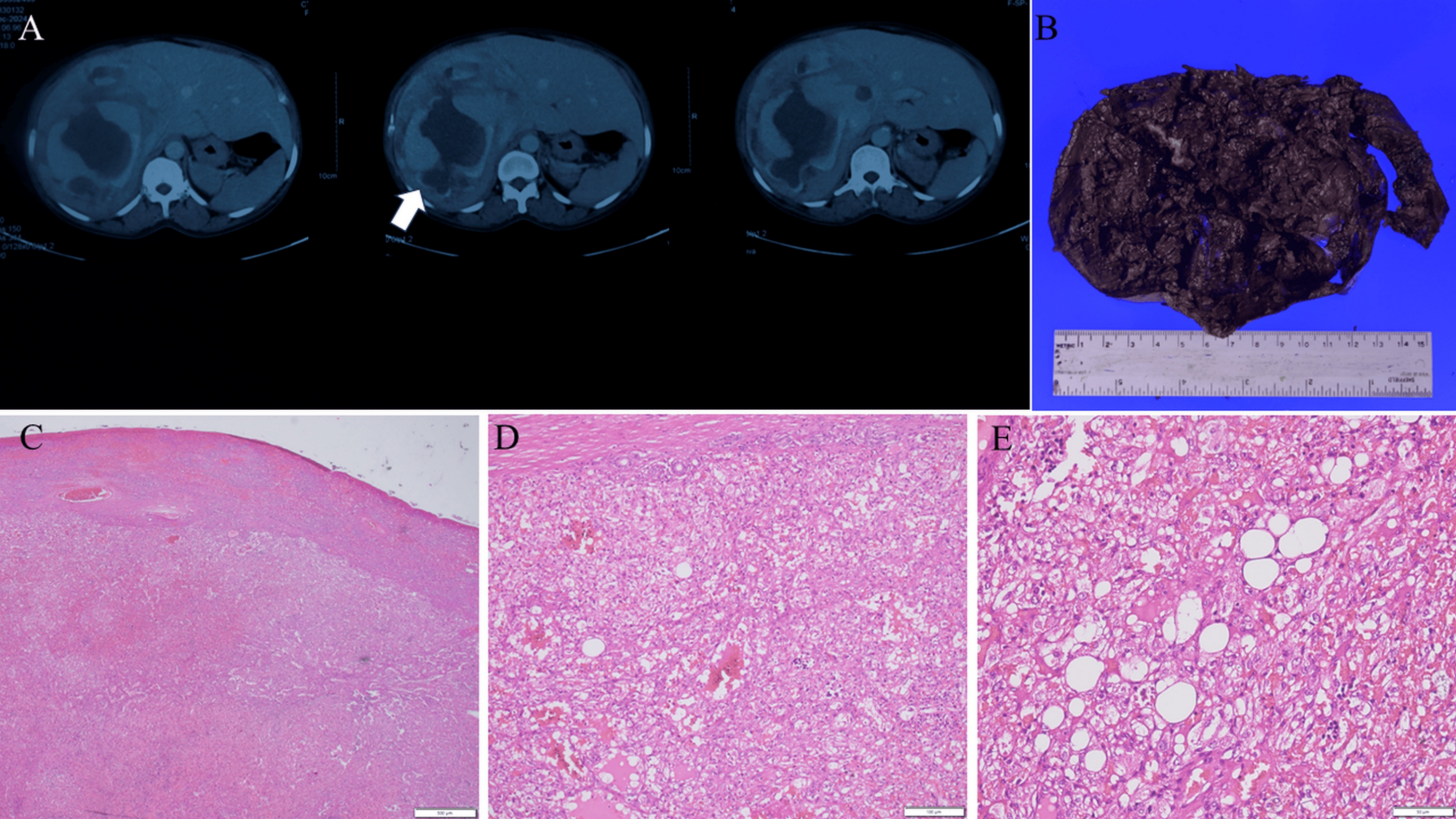
Radiology, gross examination, and histopathology CECT of the liver highlighting a well-defined, heterogeneous, enhancing mass in the right lobe of the liver (thick white arrow) (a); gross image of the resected mass showing a fleshy, friable, tan-brown mass (b); histopathology of the mass highlighting a partially encapsulated mass (c) composed of epithelioid cells (d), spindle cells, and adipocytes (e) (hematoxylin and eosin; scale bars attached). CECT: Contrast-enhancing computed tomography.

Triphasic contrast-enhancing computed tomography (TP-CECT) revealed a well-defined heterogeneous enhancing mass in the right lobe of the liver (entire segment 7 with marginal involvement of segments 6 and 8) measuring 16.4 x 15.2 x 15.3 cm (Figure 1).

Solid components of the mass showed heterogeneous arterial enhancement, which increased gradually in the portal venous and delayed phases. The patient underwent right hepatectomy, including segments 5, 6, 7, and 8, along with cholecystectomy.

Case 2

The second patient was a 38-year-old man who presented with weakness and weight loss for five months. He had mild anemia and mildly elevated serum aminotransferase levels (Table 1). His serum AFP, CEA, and CA-19-9 levels were normal. TP-CECT revealed a well-defined, heterogeneous mass lesion in the left lobe of the liver measuring 12 × 7.5 × 11.7 cm with an exophytic bulge. The arterial phase showed heterogeneous enhancement with central non-enhancing areas and progressive centripetal filling during the subsequent venous and delayed phases. The patient underwent left hepatectomy.

Histopathology and Immunohistochemistry of Both Cases

**Figure 2:**
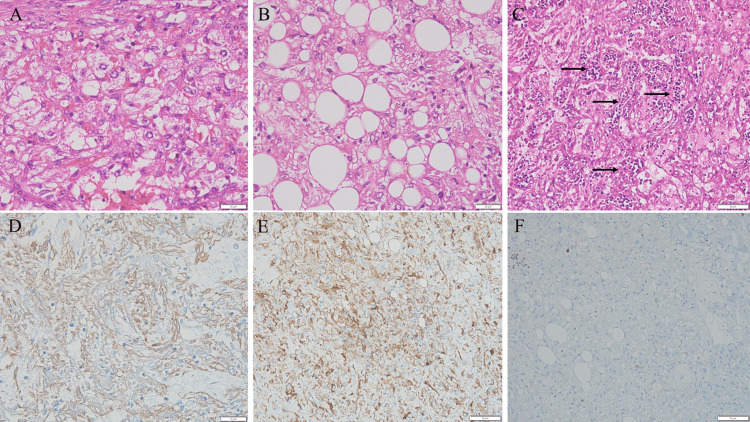
Histopathology and immunohistochemistry The epithelioid cells highlight a spider-web appearance (a), whereas the spindle cells depict short fascicles with interspersed mature adipocytes (b). Numerous erythroid colonies (thin black arrows) were noted within the sinusoid-like spaces (c) (Hematoxylin and eosin). Immunohistochemistry for SMA (d) and HMB45 (e) was positive, whereas the Ki67 index was <1% (f) (scale bars attached). SMA: Smooth muscle actin.

Both specimens showed well-circumscribed tumors with fleshy, friable cut surfaces, tan-brown to dark-brown in color, and multiple cystic/hemorrhagic areas. The peripherally preserved thin rim of native hepatic parenchyma appeared non-cirrhotic in both cases (Figure 1). Histopathological examination of the masses showed partially encapsulated tumors composed of a variable admixture of spindle cells (60% and 5%), epithelioid cells (30% and 90%), mature adipocytes (10% and 5%), and thick-walled blood vessels (Figure 1). The epithelioid cells showed diffuse sheet-like and trabecular arrangements with a polygonal shape, abundant clear cytoplasm, and an occasional “spider-web” appearance due to central cytoplasmic clumping. The spindle cells showed a short fascicular arrangement with wispy eosinophilic to vacuolated cytoplasm. Many mature adipocytes were randomly distributed. Multiple foci showed thick-walled vascular channels. Extramedullary hematopoiesis, highlighted by colonies of erythroid cells, myeloid cells, and/or occasional megakaryocytes, was noted in the first case (Figure 2).

The second case lacked extramedullary hematopoiesis but highlighted occasional intracytoplasmic melanin pigment. Mitoses were infrequent (<1/50 HPF). Cystic degeneration and hemorrhage were present​​​​​, although there was no necrosis.

Both the epithelioid and spindle cell components were positive for smooth muscle actin (SMA) and melanocytic markers (HMB45 and Melan-A), and negative for pancytokeratin, S100, Heppar-1, arginase, CD34, ERG, and c-kit. The Ki-67 index was <1% (Figure 2), and the TFE3 immunostaining was negative. A final diagnosis of hepatic angiomyolipoma (HAML) was rendered in both cases. Both patients had uneventful recoveries and were doing well at seven and four months after the surgery, respectively.

## Discussion

Hepatic AMLs (HAMLs) are incidental lesions. In a large series, more than three-fourths of HAML patients were asymptomatic, whereas abdominal discomfort and weight loss occurred in merely 15.2% and 1.7% of the patients, respectively [4]. Other presenting features include visceromegaly, nausea, and diarrhea. The presence of anemia in both patients could be related to intratumoral hemorrhage, although other causes, such as iron-deficiency anemia, were possible. The patient was not evaluated for anemia at the time of surgery. HAMLs occur across a wide age range, with a mean/median age in the fourth decade of life. A female preponderance was observed in the previous series [1,4]. Radiology is often non-diagnostic, and diagnostic accuracy depends on lesion composition and the radiologist's experience. In a study, only 3.7% of cases had a correct preoperative diagnosis due to nonspecific radiologic features [7]. CT and MRI are better modalities than ultrasonography, which usually depict hyperechoic foci [4].

The markedly elevated PIVKA-II level in the first case is a unique feature. PIVKA-II is a serum biomarker used for the detection, surveillance, treatment response, and prognosis of hepatocellular carcinoma (HCC). It provides a superior diagnostic performance in conjunction with AFP than AFP alone. PIVKA-II values are unaffected by chronic viral hepatitis or advanced liver disease, unlike AFP, although PIVKA-II elevation is reported in alcoholic liver disease, acute liver failure, vitamin K deficiency of any cause, administration of drugs such as warfarin or antibiotics, and in association with elevated serum bilirubin or ALP [8]. There was only an occasional previous report documenting PIVKA-II elevation in HAML [7]. The index case did not show any of the abovementioned features to explain the elevation of PIVKA-II. Thus, the reason for the elevated PIVKA-II in the first case remains elusive, although it can be an uncommon diagnostic pitfall in HAML that mimics HCC. The marked elevation of PIVKA-II in the first case, in conjunction with a giant, hypervascular mass on CT, demonstrates how the tumor can completely mislead the clinician before surgery.

Classical histomorphology of HAML includes a variable admixture of epithelioid cells, spindle cells, and adipocytes with or without interspersed thick-walled vascular channels. Tsui et al. subcategorized the tumor into mixed, lipomatous (≥70% fat), myomatous (≤10% fat), and angiomatous types, with the mixed type being the most common [1]. Hematopoietic elements are more commonly noted in HAML than in renal AML. They were noted in approximately 75% of HAML cases in one study [2]. The presence of hematopoietic elements in HAML could be related to the hepatic sinusoidal microenvironment, hepatic stress, hypoxic induction, or the tumor microenvironment [1]. Hematopoietic elements may be an important diagnostic clue for distinguishing HAML from other sarcomas. The presence of intracytoplasmic melanin, intratumoral hemosiderin, or both had been observed in previous studies [1,2]. Melanin pigment is not consistently observed in all cases of HAML, but when present, it reinforces the myomelanocytic nature of these tumors. The presence of this pigment is an important diagnostic clue, although it can occasionally be confused with metastatic melanoma, especially in small biopsies.

The histomorphology of HAML is variable and differs from case to case, depending on the relative proportions of epithelioid cells, spindle cells, adipocytes, and vasculature. The HAML with a predominant population of epithelioid cells morphologically resembles hepatocellular carcinoma, other primary or metastatic carcinomas with clear-cell morphology, epithelioid gastrointestinal stromal cell tumor (GIST), epithelioid hemangioendothelioma (EHE), epithelioid angiosarcoma, metastatic melanoma or clear-cell sarcoma, epithelioid leiomyosarcoma, epithelioid follicular dendritic cell sarcoma, epithelioid malignant peripheral nerve sheath tumor, and other sarcomas with epithelioid morphology [5-7,9,10]. In contrast, the one with a dominantly spindle cell or dual epithelioid-spindle cell component simulates sarcomatoid hepatocellular carcinoma, other sarcomatoid carcinomas, metastatic melanoma, gastrointestinal stromal cell tumor, solitary fibrous tumor, leiomyosarcoma, and other sarcomas [5-7,9-11]. The distinction between these entities is often based on histopathology along with a panel of immunohistochemical markers. Carcinomas usually express pancytokeratin, whereas HAML does not. The hepatocellular differentiation markers, such as HepPar1 and arginase, are also not expressed in HAML. Various other tumors express specific immunomarkers, including c-kit and DOG1 in GIST, ERG in EHE and angiosarcoma, S100 in neural tumors, STAT6 in solitary fibrous tumor, and CD21 and CD23 in follicular dendritic cell sarcoma. Leiomyosarcoma expresses SMA, desmin, h-caldesmon, and other smooth muscle markers but does not express the melanocytic markers. In contrast, melanoma or clear-cell sarcoma expresses melanocytic markers such as HMB45, Melan-A, and MART1 but does not usually express smooth muscle markers [5-7,9-11]. Occasionally, the morphology resembles a hepatic lipoma, another uncommon entity with only a few interspersed cells showing myomelanocytic differentiation [1]. Thus, histological documentation of all components within the same lesion, supplemented by judicious use of immunohistochemistry, is often sufficient for the diagnosis of HAML; the HMB45- and SMA-dual-positive cells within the lesion serve as the diagnostic pivot.

Most HAMLs run a benign course, and surgery is often curative. Approximately 10% of HAMLs show recurrence or metastasis [7]. Recurrence or metastasis often depends on larger size, nuclear atypia, pleomorphism, mitosis, and necrosis [12]. A subset of HAML and other PEComas can harbor loss-of-function mutations in TSC1/TSC2, components of the PI3K/AKT/mTOR pathway. A smaller subset can also show TFE3 gene rearrangements. The dysregulation of the mTOR pathway often explains the angiogenesis in these tumors. The molecular profile of PEComas has prognostic significance. Mutations in TSC1/TSC2 and dysregulation of mTOR are usually associated with a more indolent disease course. An aggressive behavior (e.g., metastasis) may involve additional genetic alterations, including alterations in the p53 pathway. Thus, mTOR inhibitors, such as sirolimus or everolimus, can be used in treating advanced, unresectable, or metastatic HAMLs [7,13,14].

## Conclusions

HAML is an uncommon mesenchymal neoplasm of the liver. The diagnosis of HAML depends on histopathological evaluation and documentation of myomelanocytic differentiation in the tumor cells by immunohistochemistry. The clinician and the pathologist should be aware of this entity as it is an uncommon tumor and often mimics other epithelial malignancies, including hepatocellular carcinoma. Awareness of histomorphology and judicious use of immunohistochemical markers are necessary to establish the correct diagnosis in small biopsies and resection specimens.
